# International initiatives to enhance awareness and uptake of open research in psychology: a systematic mapping review

**DOI:** 10.1098/rsos.241726

**Published:** 2025-03-19

**Authors:** Magda Skubera, Max Korbmacher, Thomas Rhys Evans, Flavio Azevedo, Charlotte R. Pennington

**Affiliations:** ^1^ School of Psychology, Aston University, Birmingham, UK; ^2^ Mohn Medical Imaging and Visualisation Centre, Bergen, Norway; ^3^ Department of Neurology, Haukeland University Hospital, Bergen, Norway; ^4^ School of Human Sciences and Institute for Lifecourse Developments, University of Greenwich, London, UK; ^5^ Department of Interdisciplinary Social Sciences, Utrecht University, Utrecht, The Netherlands

**Keywords:** open research, open science, initiatives, research reform, systematic review

## Abstract

Concerns about the replicability, reproducibility and transparency of research have ushered in a set of practices and behaviours under the umbrella of ‘open research’. To this end, many new initiatives have been developed that represent procedural (i.e. behaviours and sets of commonly used practices in the research process), structural (new norms, rules, infrastructure and incentives), and community-based change (working groups, networks). The objectives of this research were to identify and outline international initiatives that enhance awareness and uptake of open research practices in the discipline of psychology. A systematic mapping review was conducted in three stages: (i) a Web search to identify open research initiatives in psychology; (ii) a literature search to identify related articles; and (iii) a hand search of grey literature. Eligible initiatives were then coded into an overarching theme of procedural, structural or community-based change. A total of 187 initiatives were identified; 30 were procedural (e.g. toolkits, resources, software), 70 structural (e.g. policies, strategies, frameworks) and 87 community-based (e.g. working groups, networks). This review highlights that open research is progressing at pace through various initiatives that share a common goal to reform research culture. We hope that this review promotes their further adoption and facilitates coordinated efforts between individuals, organizations, institutions, publishers and funders.

## 1. Introduction

*Is there currently a crisis of confidence in psychological science reflecting an unprecedented level of doubt among practitioners about the reliability of research findings in the field? It would certainly appear that there is*.—[1, p. 528]

Concerns regarding the replicability, reproducibility and transparency of psychological research have proliferated in recent years, sparking what is now referred to commonly as the ‘replication crisis’.^1^ Despite similar concerns being debated passionately in the 1960s (see [6]), and not being exclusive to psychology [7,8], a series of landmark events in the 2010s has led to fast-paced action aiming to reform this discipline (see [3]). One event was the publication of Bem [9] who across nine experiments reported evidence of precognition—a phenomenon which proposes that people’s conscious awareness of future events can influence current ones. Surprised by how these findings could be published, many researchers voiced concerns about the inherent flexibility involved in the process of designing and analysing scientific studies, with such ‘researcher degrees of freedom’ likely leading to a prevalence of false positives in the published literature [10,11]. Independent teams of researchers subsequently failed to replicate Bem’s findings [12,13]. Around the same time, a high-profile case of academic fraud was proven in psychology, with Diederik Stapel admitting to fabricating data across many of his publications [14]. These events led to a special issue of *Perspectives on Psychological Science* on replicability in psychological science, with Pashler & Wagenmakers [1] asserting that the discipline was facing a ‘crisis of confidence’.

Yet without replication as the norm in psychology [15], the extent of this crisis remained relatively unknown until the conclusion of a 3 year large-scale replication project led by the Open Science Collaboration (OSC) in 2015 [16]. In a mammoth effort including over 270 international researchers, the OSC aimed to replicate 100 randomly selected findings from three prestigious psychology journals, finding that only 36% successfully replicated with a statistically significant effect in the same direction as the original study, and effect size estimates 32% smaller. Had the original effects been true, a minimum replication rate of 89% would have been expected [17]. This high proportion of ‘failures’ to replicate is consistent with accumulating evidence from other replication studies, despite the use of well-powered samples, preregistered protocols, tests of moderators and exploration of variation across samples and settings [18–20]. The discipline of *meta-science—*the scientific study of science itself—has shed light on many intertwining contributors to low replicability, reproducibility and transparency in research [21]. For example, researchers have outlined numerous questionable research practices (QRPs), such as hypothesizing after the results are known (HARKing) [22], and *p*-hacking techniques that exponentially increase the likelihood of detecting false positives [10,23–25]. Furthermore, academic incentive structures have received greater critical revaluation for their focus on quantity over quality, arguably contributing to weak specification of theories and analysis plans, inadequate statistical power, poor measurement, a lack of replication and reproducibility checks, and non-transparent reporting (see [26,27]). Many biases also influence both individual researchers and the wider research landscape, such as confirmation bias whereby researchers favour evidence in line with their expectations, and publication bias whereby journals value positive over null or inconclusive findings [4,28]. Together, such incentives have built a research ecosystem that has rewarded and recognized the wrong elements of research—the novelty of results over robust and transparent methods and inferences.

Optimistically, through a better understanding of these issues, a new era of ‘open research’ has been fast advanced with the goal of reforming research and the more general ecosystem in which it sits. Open research, also referred to as open science or open scholarship, is an umbrella term reflecting the idea that ‘scientific knowledge of all kinds, where appropriate, should be openly accessible, transparent, rigorous, reproducible, replicable, accumulative and inclusive’ [29]. Within this sphere, many practices, such as preregistration, registered reports (RRs), open materials, code, and data, and article preprints have been developed and/or re-ignited across the research pipeline. Study preregistration allows researchers to initiate a time-stamped plan of their research questions, hypotheses, methods and analysis plan prior to data collection and/or analysis, and is proposed to enhance transparency, limit analytical flexibility (or make it more detectable), and allow others to transparently evaluate the capacity of analyses to falsify a prediction (see [30–32] for various perspectives). Importantly, preregistration can be implemented for all kinds of research (e.g. primary and secondary data analysis; qualitative and quantitative [27,33,34]) and represents a ‘plan and not a prison’ whereas necessary deviations can be documented [35]. RRs represent a publishing model that integrates preregistration; in a ‘Stage 1’ protocol, researchers submit their research question(s), hypotheses and detailed methods and analysis plans for peer review, and if this protocol is deemed to meet the RR criteria, a decision of ‘in principle acceptance’ is offered. At ‘Stage 2’, the researchers then append their results and discussion, and final acceptance is based on adherence to the Stage 1 protocol and the accurate representation of the results. Unlike traditional articles, then, RRs shift the focus to rigorous methodology and analytical reporting rather than the nature of the results [36]. Another practice of making all study materials (e.g. survey items, stimulus materials), code (programming and analysis) and data publicly available facilitates replication, reproducibility and reuse [37–39]. At the point of dissemination, there has been greater adoption of open access publishing [40] with preprint servers (e.g. PsyArXiv) representing green open access repositories, thus allowing free access to research, earlier discoverability, faster feedback and correction mechanisms [41].

By way of improving wider research culture, there have also been initiatives to foster better equity, diversity, inclusion, accessibility and representation in psychological science, in terms of researchers, early career scholars and study participants (see [42–46]). For example, there have been efforts to improve collaborations across the Global North and South to advance scientific knowledge (e.g. [47,48]), and an increased recognition of ‘citizen science’—directly involving members of the general public in research [49]. Organizations such as the Framework for Open & Reproducible Research Training (FORRT) aim to bridge open research through open education, pedagogical reform and social justice advocacy to foster inclusive and participatory research practices across diverse geographies, disciplines and contexts. As such, open research not only aims to foster research integrity but build a more inclusive scientific community to accelerate solutions for complex problems and democratize knowledge [50].

**Figure 1.**
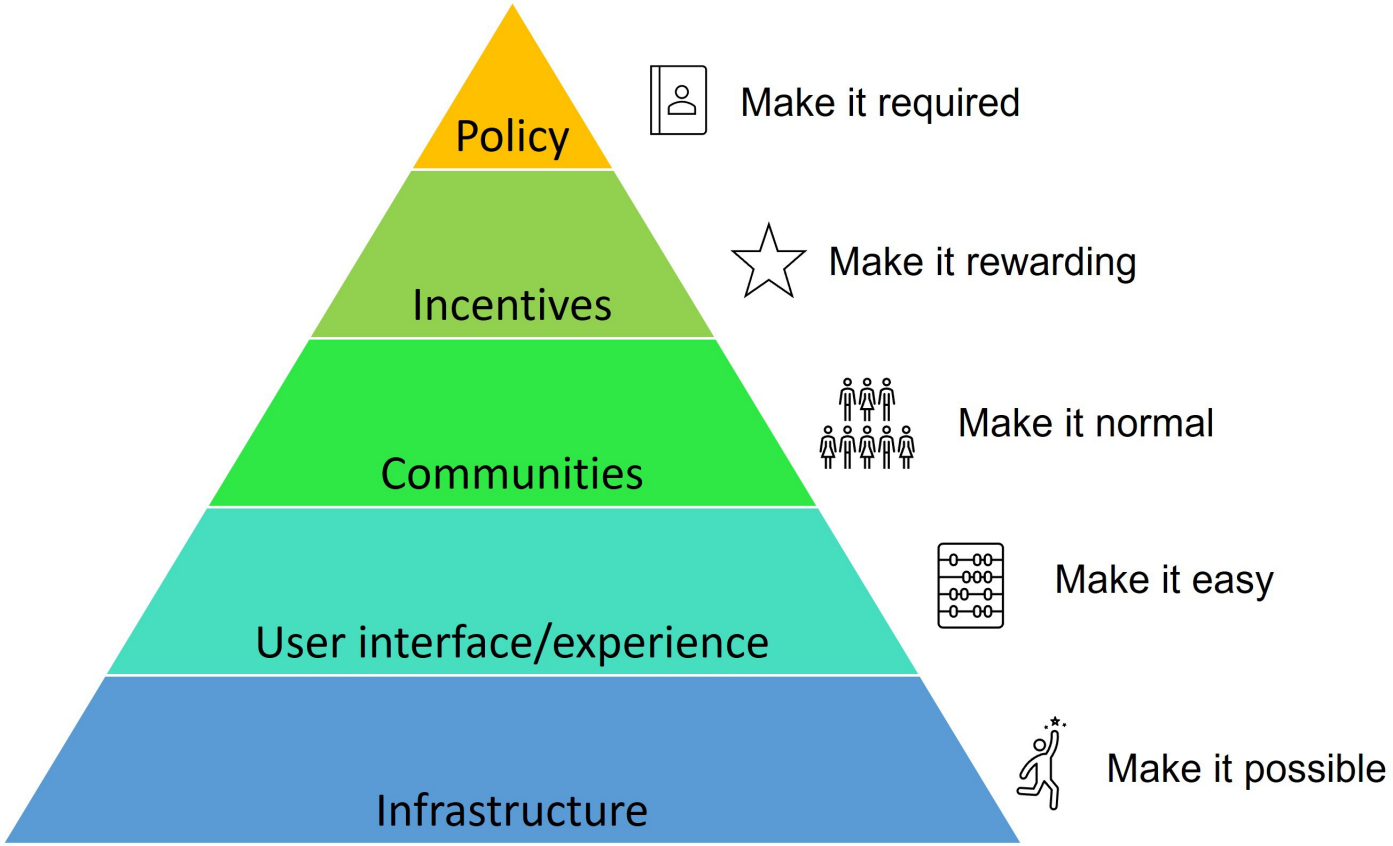
The Center for Open Science (COS) strategy for culture change. Note that this figure has been reproduced from https://www.cos.io/blog/strategy-for-culture-change under a CC-BY-4.0 licence.

To facilitate wide-scale and permanent uptake of open research, however, we need to focus on behaviour change—and behaviour change is hard. Researchers are embedded within a historical social and cultural system which shapes their behaviour through the communication of norms (this is what we do, this is what other researchers should do), the power of incentives (this is what researchers are rewarded for), and the integration of current policy (this is what a researcher needs to do as part of the system) [51]. With this in mind, different strategies for culture change have been progressed, such as the Center for Open Science’s strategy to make it possible, easy, normative, rewarding and required (see figure 1; with the caveat of this being where possible and appropriate). Each of these elements will depend on various initiatives created by groups of people, research institutes, organizations, publishers and funders. Indeed, many different open research initiatives are developing at pace, with a recent commentary outlining several procedural (i.e. behaviours and sets of commonly used practices in the research process), structural (new norms, rules, infrastructure and incentives) and community changes (teamwork and collaboration [52]) that can be mapped onto the aforementioned strategy of behaviour change. However, for such initiatives to be useful and effective, we need to ensure that we are not duplicating efforts at the risk of creating fragmented (and overworked) communities. In other words, reforms to improve research culture must be coordinated across the ecosystem, including individuals, research groups, journals, funders and institutional bodies. One first step is to identify and outline the various open research initiatives that currently exist to foster awareness and uptake of open research and facilitate further collaborative efforts.

To this end, we performed a systematic mapping review to identify and outline international initiatives that enhance awareness and uptake of open research practices in the discipline of psychology. Following the PRISMA guidelines for systematic reviews, we first conducted an extensive webpage search to identify different initiatives that may not be described in the published literature. We then conducted an empirical literature and hand search to identify articles citing the initiative that provided further information about its goal and scope, as well as to identify any additional initiatives. In line with a recent commentary by Korbmacher *et al*. [52], we categorized each initiative into one of three themes: procedural, structural or community change. We focused exclusively on the discipline of psychology because it has been a trailblazer for many new open research initiatives owing to its so-called ‘replication/reproducibility crisis’ and can therefore provide a roadmap for other disciplines that are experiencing, or are yet to experience, similar issues. However, it is important to note from the start that some of the initiatives identified have already been adopted across research disciplines and many could be implemented to foster long-term, sustained behaviour change. As such, this review will be useful across disciplines aiming to increase awareness and uptake of open research.

## 2. Method

### 2.1. Literature search

This systematic mapping review was conducted in line with the 2020 Preferred Reporting Items for Systematic Reviews and Meta-Analyses Statement (PRISMA) [53]. The PRISMA 2000 checklist, screening, article records and supporting materials for this review are available at: https://osf.io/uap7j/.

### 2.2. Search strategy

The review comprised three stages. In Stage 1, we conducted an online Web search of open research initiatives based on the rationale that not all initiatives would be available in the published literature (i.e. university-level initiatives, podcasts, etc.). In Stage 2, we conducted an empirical literature search to bolster this Web search with any associated articles that describe or provide an overview of the identified initiatives and to identify additional initiatives missed in Stage 1. The keywords and search terms were: *open research/science/scholarship initiatives; resource; guidelines; strategy; agenda; policy; schemes;* and *organizations* using the following Boolean operators:

(((Open (research OR science OR scholarship) AND (initiatives OR resource OR guideline OR strategy OR agenda OR policy OR schemes OR organisation)))) AND Psychology

In Stage 3, a hand search of initiatives was conducted via Google to identify initiatives that the authors were aware of but that were not identified in Stages 1 and 2 and/or associated grey literature that was not yet published (e.g. preprints). For the latter, this was conducted by searching for the initiative name using the electronic databases stated below.

The Stage 1 Web search was conducted using Google between 16 May 2023 and 2 August 2023 and involved searches of international institutional, government and organization Web pages (e.g. Open Science Framework; Framework for Open & Reproducible Research Training), as well as social media platforms such as X and Facebook where open research initiatives are routinely described and promoted. The Stage 2 search was conducted on 29 August 2023 and involved a literature search of articles from electronic databases, specifically the Web of Science Core Collection (Clarivate 2024™), Scopus, EBSCOhost and PubMed, and the psychology-specific preprint server PsyArXiv. Additional filters included a date range of 1 January 2011 to 29 August 2023 and the inclusion of ‘psychology’ in the search strategy to exclude non-psychology articles, as well as the exclusion of review articles excluded via tick boxes. The year 2011 was selected as the start date because it represents the year in which several notable controversies (see [11,27,54]) sparked debate of a ‘replication crisis’ in psychology [1], and led to a paradigmatic shift towards open research [4,28,55]. The Stage 3 hand search was conducted between 1 April 2024 and 1 June 2024 during drafting of this paper.

### 2.3. Eligibility criteria

The inclusion criteria for all stages were as follows:

1. Web pages and/or literature articles that describe open research initiatives.
2. Initiatives established between the date range of January 2011 and June 2024.
3. Eligible resources that were relevant to the discipline of psychology, by either referencing psychology specifically or other relevant fields such as the social sciences, neuroscience, research or science.
4. Initiatives that were written in or translated to English language (due to the coder’s native language).
5. Initiatives that were fully established (i.e. not in the conception or development phase, not retired) and original published articles (i.e. no review articles).

### 2.4. Screening procedure

All data were reviewed by the first author (M.S.) and verified by the lead author (C.R.P.). The search results from each stage were input into a Microsoft Excel spreadsheet after which duplicates were removed by sorting the references alphabetically. In Stage 1, relevant initiatives were identified via a Web search with their Web page reviewed against the inclusion criteria. In Stage 2, published articles were identified that either supported an initiative identified in Stage 1 or identified a missed initiative. Here, the abstract of each article was reviewed against the inclusion criteria after which a full-text review was performed. In Stage 3, a hand search was conducted to identify any articles relating to an already identified initiative and this was then added alongside the original in the spreadsheet. All exclusions are outlined in table 1.

**Table 1.** Exclusion criteria and reasons.

exclusion criterion	reasons
not an initiative	the webpage or article does not outline or discuss an initiative: it discusses open research generally (e.g. definitions, commentaries)
initiative not current	the open research initiative is not fully established, may have been piloted, or retired
initiative prior to 2011	initiative established prior to 2011 before the advent of the ‘replication crisis’ in psychology
not open research focused	the initiative is not related to, or does not focus on, open research; for example, it vaguely mentions open research practices used in articles, but not with the aim of increasing awareness or uptake of open research
not in the English language	the webpage or article describing the initiative is not written or translated into the coders’ native language of English
not within the discipline of psychology	the focus of the article or initiative is not relevant to the discipline of psychology. For Stage 1, this includes initiatives that are specifically related to another discipline and are not related to or could be used within psychology. For Stage 2, this includes articles that make broad reference to psychology but implement the initiative in a different discipline (e.g. an initiative from pharmacy that discusses psychological effects)
not a literature article	conference presentations, corrections to previous articles, theses and dissertations were excluded. However, conference proceedings (full academic papers published in the context of an academic conference or workshop) were eligible for inclusion
not an additional initiative (Stage 2)	articles that discuss an initiative that was already identified in the Stage 1 Web search and had a supporting publication
review article (Stage 2)	articles that are a review article synthesizing open research initiatives (e.g. other narrative or systematic reviews and meta-analyses)
full text not available (Stage 2)	no full text of the article is available
unable to access full text (Stage 2)	unable to access full text through institutional or public access platforms (e.g. institutional repositories, preprint servers, Google Scholar, ResearchGate)

### 2.5. Quality assessment

A quality assessment was not required for this systematic review because no evaluation was undertaken; specifically, this review mapped current initiatives with a focus on initiative foci and not efficacy. The landscape is currently lacking robust evaluation because it is very dynamic and still in its infancy (see [56]). As such, we do not judge open research initiatives to be of high or low quality because they are simply intended to increase awareness and/or uptake. This review therefore identifies and outlines open research initiatives within psychology with the goal of facilitating their adoption and wider research culture reform where relevant and appropriate.

### 2.6. Data extraction

The Web and literature searches were conducted, and the data retrieved by the first author (M.S.). In Stage 1, the following details were recorded in Microsoft Excel: initiative name, description, country of origin, stage initiative was found, associated articles, link/URL to the initiative webpage and the coded thematic category (see §3 below and https://osf.io/uap7j/ for supporting information). In Stage 2, a search of existing literature was conducted to identify articles citing the initiatives found in Stage 1 and identify any that were missed; any initiative that did not already have an associated article then had this inserted alongside it in the ‘Articles Citing Initiatives’ cell. In Stage 3, a hand search was conducted to identify any initiatives that were not identified in Stage 1 and 2, as well as any associated articles. The cell ‘Stage Found’ states whether the initiative was found at Stage 1, 2 or 3.

### 2.7. Data synthesis and analysis

The analysis strategy follows a narrative synthesis approach using the guidelines outlined by Popay [57]. This approach uses text to synthesize or ‘tell the story’ of findings and is appropriate when statistical data are not used. Using the initiative’s current description, and focusing on its primary goals, the first author coded each to one of three thematic categories of *procedural*, *structural* or *community change* for narrative synthesis, in accordance with [52]. Table 2 provides the definition for each theme which guided this categorization. The lead author (C.R.P.) then masked coded these initiatives to the same three themes and any discrepancies were reviewed by two additional coders (T.R.E. and M.K.) and then agreed upon by the entire project team. As this review does not rely on statistical data, there was no requirement for a method to handle missing data nor an assessment for risk of bias due to missing results. There were also no requirements for an assessment of certainty of the body of evidence because the nature of this review is to map out open research initiatives that aim to enhance awareness and uptake of open research in psychology.

**Table 2.** Definitions of thematic categories.

theme	definition
procedural initiatives	*procedural initiatives encompass behaviours and sets of commonly used practices in the research process* i.e. Initiatives that help researchers to use open research practices or change behaviours to improve the research landscape (e.g. guidebooks, toolkits, code, templates, Web platforms, datasets/bases, etc.)
structural initiatives	*initiatives that describe and outline new norms and rules at the institutional level, create new infrastructure, or embed open research practices into educational curriculum and/or incentivize researchers to adopt improved practices* i.e. Initiatives typically at a structural/top-down level that make possible, embed or even mandate the uptake of open research practices, across research institutions, groups or organizations
community initiatives	*community initiatives foster teamwork, collaboration and discussion within the scientific community to increase awareness or uptake of open research practices* i.e. These initiatives are usually bottom-up, grassroots initiatives (e.g. led by students, early career researchers) that aid awareness and uptake of open research through supporting, promoting and community building

## 3. Results

### 3.1. Search yield

The Stage 1 Web search yielded a total of 315 initiatives and the Stage 2 literature search identified 2809 articles (Web of Science: *n =* 2243; EBSCO: 252; PubMed: 91; Scopus: 39; PsyArXiv: 184). Before the abstract review, 2 duplicates in Stage 1 and 179 duplicates in Stage 2 were removed. Stage 3 yielded an additional 15 initiatives and 104 additional articles.

In the abstract review, 161 initiatives in Stage 1 and 2396 articles in Stage 2 were removed for the reasons articulated in table 1. The reasons were as follows: not an initiative (Stage 2, *n* = 256), initiative not current (Stage 1, *n* = 10), initiative prior to 2011 (Stage 1, *n* = 47; Stage 2, *n* = 256), not open research focused (Stage 1, *n* = 38; Stage 2, *n* = 453), not in English language (Stage 1, *n* = 3; Stage 2, *n* = 1), not within the discipline of psychology (Stage 1, *n* = 60; Stage 2, *n* = 1409) and not a literature article (Stage 1, *n* = 3; Stage 2, *n* = 21).

In Stage 2, the remaining 334 texts were fully screened by reading their full text and 290 were excluded as follows: not an initiative (*n* = 81), prior to 2011 (*n* = 16), not open research focused (*n* = 13), not in English language (*n* = 34), not within the discipline of psychology (*n* = 42), not an additional initiative (*n* = 72), review article (*n* = 17), no full text available (*n* = 14) and unable to access full text (*n* = 1). After these exclusions, the remaining 44 articles were included of which 22 articles provided initiatives that were not identified in Stage 1 and 22 provided an article associated with initiatives already identified in Stage 1.

**Figure 2.**
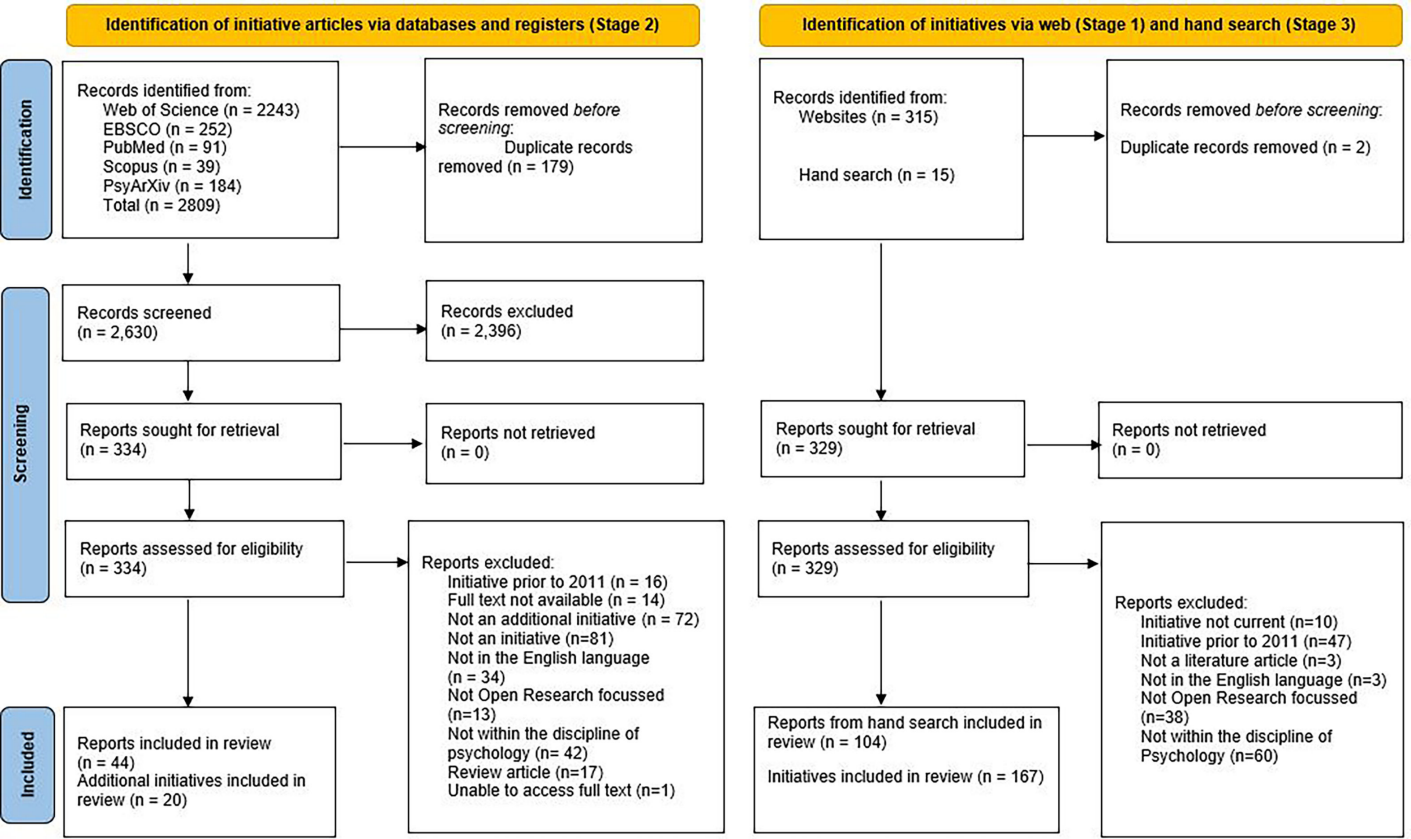
PRISMA flow diagram.

In Stage 1 (Web search), there remained 152 initiatives, Stage 2 (literature search) provided 20 new initiatives and Stage 3 (hand search) provided 15 new initiatives. The total number of initiatives included for the review was 187. Of these, 30 were coded as procedural, 70 structural and 87 community based. Figure 2 provides the PRISMA flow diagram and electronic supplementary material, table S1, provides a detailed breakdown of each initiative. In the review below, we provide direct links to each initiative and the associated articles. With regards to the latter, it is important to note that the citations are not always the authors and/or developers of the initiative: that is, our systematic search also identified articles that describe or outline each initiative.

### 3.2. Procedural initiatives

Toolkits and resources help researchers, publishers, universities, research organizations and other stakeholders understand how to use different open research practices, and their associated platforms provide the infrastructure to house them. A total of 30 procedural initiatives were identified that provide such toolkits, guidebooks, templates, tools, datasets and web applications.

Some platforms and toolkits provide support and platforms for specific open research practices, such as preregistration and open data. The platform AsPredicted [3,58] was launched in 2015 and makes it easy for researchers to preregister their studies and allow other researchers to read, verify and evaluate them. Here, researchers answer nine simple questions regarding their study and, once submitted, a time-stamped PDF document is registered to the domain. As of now, preregistration is not mandated by most journals, funders, institutions or research organizations, with AsPredicted providing the tools and infrastructure for researchers to register their studies if they choose. Other platforms also facilitate open research practices, such as the Network of Open Science Initiatives at Psychology Departments (NOSI) [59], which provides protocols, links and resources for preprints, preregistration, RRs, open code, materials, and data, reproducible manuscripts, publishing null results, transparent qualitative research, and more. The Open Science Framework hosts guides for adopting RRs, including frequently asked questions (FAQs), resources for researchers, funders and editors, and supporting videos and articles (see also [36]).

With regards to sharing research data, OpenNeuro [60] provides a free platform for validating and sharing a broad range of brain imaging data, such as for MRI, PET, MEG, EEG and iEEG, following the FAIR principles for data sharing. The Collaborative Informatics and Neuroimaging Suite Toolkit for Anonymous Computation (COINSTAC) [61,62] is a tool developed to support federated analysis for neuroimaging data through the use of federated analysis and standardization of collaboration methods. COINSTAC enables researchers to run decentralized neuroimaging analyses to perform larger collaborative studies, enabling them to build statistical or machine learning models to advance research in this area. More generally, another initiative named OpenRefine [63] provides an open source tool for working with messy data, allowing researchers to clean and transform, and then extend it with Web services and external data. In order to facilitate the sharing of data analysis workflows, the Common Workflow Language Project [64,65] provides free and open standards for describing and sharing command-line tool based workflows to aid computational reuse and portability. It includes many features developed in collaboration with the community, such as support for software containers, resource requirements and workflow-level conditional branching. Other tools aim to improve measurement and assessment, such as the International Cognitive Ability Resourc*e* (ICAR) [66], a public-domain assessment tool to facilitate the broader assessment of neuropsychological and cognitive abilities in research and practice.

Other procedural initiatives provide open educational resources (OERs), such as training workshops, that aim to equip students and researchers with the skills to adopt open research practices. The Facilitate Open Science Training for European Researchers (FOSTER) (see [59,67,68]) provides an e-platform to host training on open research for the European community and the FOSTER Open Science Training Handbook [69] is a key educational resource for instructors and trainers that brings together methods, techniques and practices. A similar initiative, Opensciency, provides core open research curriculum through lesson plans and learning objectives to introduce students to important definitions, tools and resources in open research. The Open Science MOOC [70] provides a range of online courses to equip students and researchers with essential skills through videos, research articles, dummy datasets, code and tasks, and the LMU Open Science Center [71] provides workshop materials for study preregistration, power analysis, open data, materials, privacy and open access. The Principles and Practices of Open Research: Teaching, Research, Impact, and Learning (PaPOR TRaIL) [72] outlines a course tailored to undergraduate and master’s students to provide best scientific practice in open research and help them embed these principles and practices into their research projects.

New software has been developed to help advance open research, transparency and reproducibility. Statistical software, such as Jamovi [73,74], JASP [75,76] and R-Studio [77,78] are open-source and allow researchers to clean, screen and analyse data, create reproducible figures and tables, and share data and associated outputs that are freely accessible. Other software can facilitate the detection of errors and possible QRPs in research outputs. Specifically, P-curve [79] is a statistical tool that can be utilized to explore the evidential value of research findings or detect selective reporting from a set of quantitative findings; Stat Check [80,81] can detect statistical errors in articles by reproducing the calculations outlined in an article and highlighting inconsistencies; and Z-curve [82] provides a tool for estimating the expected replication rate of a study based on the mean statistical power after selection for significance. Together, such procedural initiatives can help researchers to embed open research practices routinely in both research and education.

There are many new initiatives that aim to improve recognition and rewards for practising open research. The Aligning Incentives Toolkit [50] was developed to support the efforts of individuals who recognize issues with the current academic rewards system and wish to address them. Through a series of fact sheets, it provides a brief overview of several topics relating to research assessment, such as aligning metrics to core values, accessibility, diversity and inclusivity, and embedding open research practices. It also includes an example worksheet on values-aligned behaviours to support research incentive reform. Similarly, NOR-CAM [83] provides a toolbox for recognition and rewards in academic careers through a flexible and holistic framework for research assessment. It includes a guide that adopts three core principles for research assessment for use by institutions, funders and national authorities: more transparency, greater breadth and comprehensive assessment. Another initiative, Ouvrir la Science, provides guidance to research organizations on how to develop and enhance their own policies and practices towards the long-term preservation and openness of research data. The UNESCO Open Science Toolkit [84] collates a set of open-access guides, policy briefs factsheets and indexes based on the UNESCO Open Science Recommendations. For example, there are guides on building capacity for open science, developing policies, funding, bolstering infrastructure, engaging societal actors and supporting open-source hardware, as well as factsheets on understanding open research and identifying predatory academic journals and conferences. Each piece is a living resource updated to reflect new developments and the status of implementation of the recommendation.

The Tools to Advance Research Assessment (TARA) is a project to facilitate the development of new policies and practices for academic career assessment. It comprises a toolkit of resources informed by the academic community to support academic institutions working to improve policy and practice as well as specific projects, such as ReformScape [71,85]—an online dataset that provides the criteria and standards that academic institutions use for hiring, review, promotion and tenure around the world. Another initiative is Project TIER [86] whose mission is to promote systemic change in professional norms related to research transparency and reproducibility. The main initiative from Project TIER is the ‘TIER Protocol’ which specifies the contents and organization of reproducibility documentation for projects involving computations with statistical data. Curate Science [87] is another initiative to strengthen research through the development of toolkits and Web applications to enhance the transparency and credibility of research. It includes a set of transparency standards, a replication tracker and transparency audits.

These aforementioned initiatives are developed mainly by researchers and organizations in the Global North (i.e. America, Europe, UK), but it is essential that developing countries are supported to embed open research within their ecosystems. The African Open Science Platform was co-founded in 2016 to convene and coordinate the interests, ideas, people, institutions and resources needed to advocate and advance open research in and for Africa. The platform’s mission is to centre African scientists at the cutting edge of contemporary, data-intensive science, signalling this as a fundamental resource for a modern society. The platform provides federated hardware, communications and software infrastructure as well as policies and resources to support open research (e.g. data management [88]). Finally, procedural initiatives have been developed to improve the publishing landscape. For example, the OAPEN Open Access Books Toolkit (see [89]) is a publicly available resource that aims to help authors better understand open access book publishing and to promote trust in open access books. The Collaborative Knowledge Foundation (CoKo) [90] is an organization that designs and builds new systems to transform and benefit the publishing community through open-source tools that enable the dissemination of critical knowledge ‘better, faster and cheaper’. They construct core open infrastructure, tools and platforms aligned with the true purpose of publishing—to advance collective knowledge.

### 3.3. Structural initiatives

Structural initiatives comprise new research frameworks, strategies, principles, policies and infrastructure that are embedded into the research ecosystem, garnering support from institutions, journals, funders and governments. A total of 70 structural-based initiatives were identified in this review.

**Figure 3.**
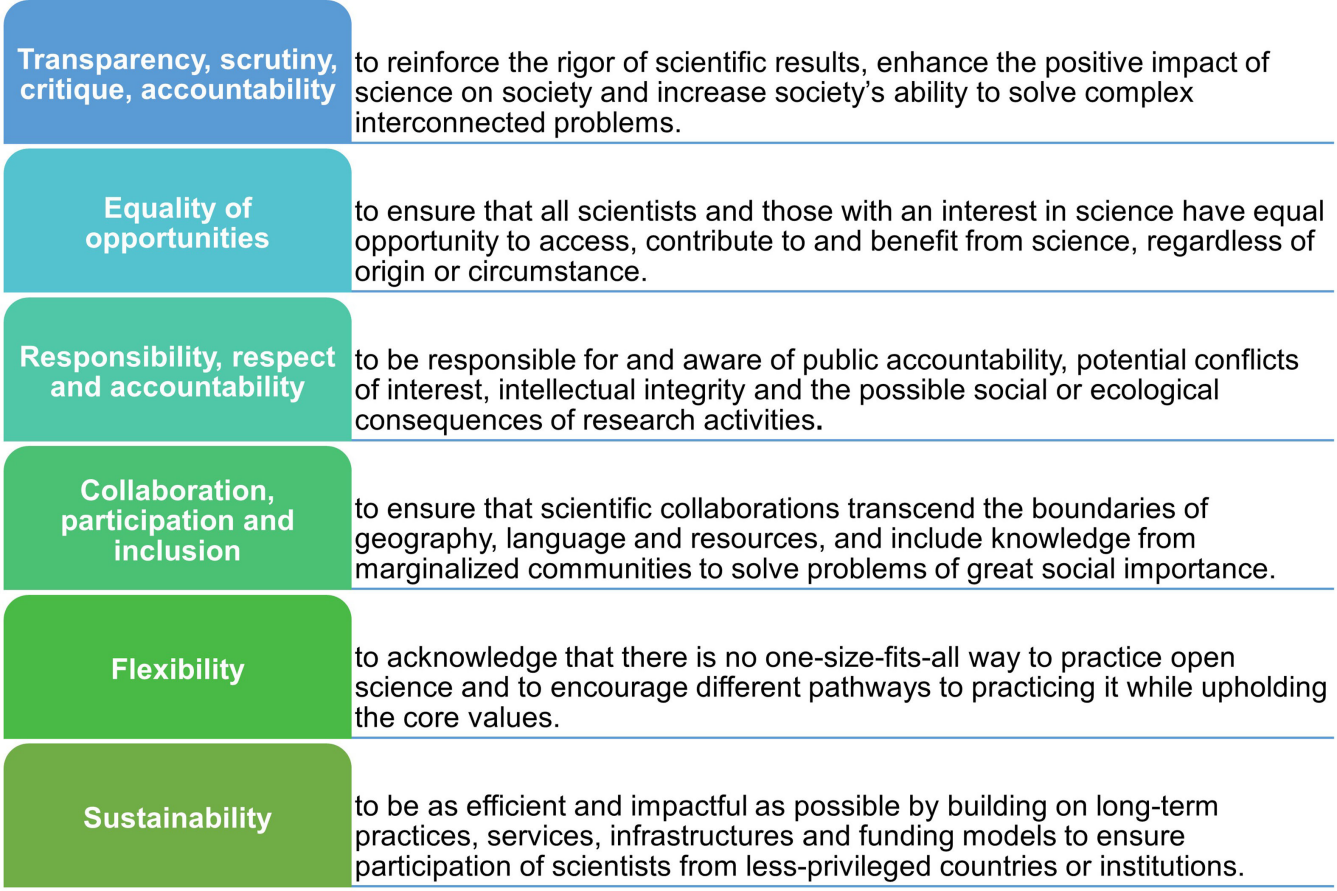
The guiding principles of the UNESCO Recommendation on Open Science. Note that this figure was produced into figure format from information provided at: https://www.unesco.org/en/open-science/about.

Many countries and nations have implemented open research agendas and policies, such as the USA’s Biden–Harris Administration (see [91]) which has initiated new grant funding, improvements in research infrastructure and expanded opportunities for research participation and public engagement to advance open and equitable research. Under this new administration, the White House Office of Science and Technology Policy (OSTP) declared 2023 the ‘Year of Open Science’, advancing many open research policies, such as a public access memorandum on ‘ensuring free, immediate and equitable access to federally funded research’ as well as the National Institute of Health’s ‘Data Management and Sharing’ policy. UNESCO, a specialized agency of the United Nations, also developed their ‘Recommendation on Open Science’ in 2021, which provides an internationally agreed definition, set of shared values and guiding principles for open research (see figure 3). It outlines a set of actions conducive to the fair and equitable operationalization of open research across individual, institutional, national, regional and international levels (see [92]). To date, over 190 countries have adopted this recommendation.

Similarly, the European University Association (EUA) has developed an Open Science Agenda for 2025 and beyond (see [93]) which defines priorities in the field of open research and describes the current context, challenges and developments. It aims to support its members to transition to open research, contributing to the development of associated policies and encouraging universities to play a more proactive role in the regulatory and financial frameworks shaping this process. The Latvian Open Science Strategy 2021−2027 [94] aims to provide society, researchers, businesses, policymakers and other stakeholders with freely accessible scientific information, and promote meaningful societal engagement in the research process. It includes several initiatives, such as requiring data management plans for all state-funded research programmes and creating an open research monitoring system. The Slovenia Scientific Research and Innovation Activities Act 2022 states that scientific research must comply with principles of open research and provides funding for the implementation of associated principles.

Other country-specific strategies include the Estonian Research and Development and Innovation Strategy 2014−2020 (see [95]), the Finnish Open Data Programme for 2013−2015 and subsequently the Finnish Open Science and Research Roadmap [96], the Croatian Open Science Cloud Initiative [97], the MINERVA project to support open science in Moldova and Armenia [98] and the Scientific and Technological Research Council’s Open Science Policy for Turkey [99]. The National Open Science Cloud Initiatives (NOSCIs) [100] is a work package of the NI4OS-Europe mission funded by the European Commission and represents a national-level coalition of open research stakeholders that seek to develop a national strategy, open services and infrastructure for open research. This initiative aims to facilitate the integration of EU Member States and associated countries in a European Open Science Cloud (EOSC)—a federated ecosystem of research data infrastructures that allows the scientific community to share and process publicly funded research across borders and scientific domains. The European Commission also envisages a strategic vision for citizen science at the national level and have developed the Mutual Learning Exercise on Citizen Science Initiatives-Policy and Practice (see [101]) that facilitates the exchange of information, experience, lessons, good practice, policies and programmes for supporting and scaling up citizen science.

Some structural initiatives focus on specific aspects of open research, such as ensuring FAIR open data and open access research outputs. The Flemish Research Data Network (FRDN) [102] has unified a network of Flemish research organizations to develop preconditions for the exchange and reuse of FAIR research (meta) data, the Turkey Research Data and Open Data Task Force has created data management plans for Turkish universities, follow world developments on open data, and support the creation of interoperable systems, and the UK’s Open Data White Paper [103] outlines how the UK will unlock and seize the benefits of responsible data sharing. The Beijing Declaration on Research Data [104] is a statement that encourages global cooperation especially for public research data. It incorporates another initiative of the FAIR principles [105,106] which provide guidelines for improving the findability, accessibility, interoperability and reuse of digital assets. The Japan Science & Technology Agency (JST) policy on Open Access [107] provides implementation guidelines for open access publications and data management. Similarly, Denmark has developed a National Strategy for Open Access, which states that from 2025 onwards there should be ‘unimpeded digital access for all peer-reviewed scientific articles from Danish research institutions’ to achieve the ‘maximum effect from research’, and Sweden has developed the Swedish Research Bill 2016/17:50 [108], which in 2016 stated that the goal is to ‘implement a full transition to Open Access to research results, including scholarly publications, artistic works and research data, within 10 years’. The Norwegian Government 2017 provided national goals and guidelines for open access to research articles, with the goal for all publicly funded Norwegian research articles to be made openly available by 2024 [109]. In 2018, the European Commission and European Research Council announced the launch of cOAlition S an initiative that, from 2021, sees all scholarly publications funded by national, regional and international research councils and funding bodies published in open access journals, on open access platforms or made immediately available through open access repositories without embargo. Funding agencies have also developed open access policies, such as the UK Research & Innovation (UKRI) funding council who mandate that research articles should be made publicly available as of 2022 and monographs and book chapters as of 2024 [110].

Such mandates have seen a rise of Green Open Access (also known as ‘self-archiving’ [111]); whereby the author’s accepted manuscript is uploaded to an institutional or disciplinary open access repository, and Gold Open Access [111], also known as paid open access, where an article processing charge (APC) is typically paid to the publisher through institutional ‘read and publish’ deals or through the researcher’s funding or expenses. New national and international policies, such as the aforementioned UNESCO recommendation on open science and cOAlition S support the development of non-commercial and community-driven forms of open access publishing, such as through Diamond Open Access [112] where outputs are preserved with no fees to either the reader or author. To accelerate *free* open access, preprint servers have been formed for many disciplines and countries, specifically AfricArXiv [113], which enhances the discoverability of research from and about Africa, PsyArXiv [75], a preprint server for the psychological sciences, and SSRN [114] and preprints.org [115] that provide a multidisciplinary platform to make early versions of research output permanently available, discoverable and citable.

New infrastructure has also been built to facilitate the implementation of open research. The Center for Open Science (COS) [116] was founded in 2013 to start, scale and sustain open research by democratizing research access, improving inclusion and diversity of stakeholders, enhancing accountability for research integrity, facilitating self-correction, and expanding transparency and sharing of all research content to improve research rigour and reproducibility. Advancing these goals, COS operates the Open Science Framework (OSF; see [117])—a free, open-source Web application that supports the entire research lifecycle from planning, execution, reporting, publishing, archiving and discovery, with OSF preprints, registries, collections and institutions. COS has also introduced the Transparency and Openness Promotion (TOP) Guidelines (see [116,118]) which are eight modular standards to move scientific communication towards greater openness: namely, citation standards, data transparency, analytic methods transparency, research materials transparency, design and analysis transparency, study preregistration, analysis plan preregistration and replication. The ‘TOP Factor’ is a metric that reports how journals adhere to these guidelines categorized as ‘not implemented’ or between Levels 1 and 3. The Research Data Alliance [119,120] is a large-scale international member-based organization focused on the development of infrastructure to reduce barriers for data sharing and exchange; it allows researchers to share and re-use data across technologies, disciplines and countries to address the grand challenges of society.

Other platforms also make sharing each element of the research cycle easier: the European Open Science Cloud (EOSC) [105,121] is an open, federated, ecosystem of infrastructure, services, research artefacts and standards that allow European researchers to engage in open research, the EOSC Future [122] is a platform for FAIR data, resources and open research services, and B2SHARE [123] enables researchers, scientific communities and citizen scientists to store, publish, explore and share FAIR-compliant data. Research repositories have also been developed by individual institutes and organizations; for example, the CeON Aggregator [124] is run by the University of Warsaw and integrates with COS to provide a single point of access for Polish repositories. The C-BIG Repository [125] was developed by the Montreal Neurological Institute to provide the infrastructure for sharing data from patients with neurological disease. The National Open Research Analytics (NORA) [126] is a Danish national initiative that provides national data infrastructure, which through Research Portal Denmark provides a national perspective on Danish research from both global, local and institutional sources. The Registry of Efficacy and Effectiveness Studies is a database of causal inference studies designed to increase the transparency of and access to information about efficacy and effectiveness studies in education and related fields (see [127]). Many new repositories have also been created in response to new research governance, support and funding policies around open science publications, such as CORE [128], La Referencia, Open Research Europe, Plan P-Transform to Open Science, the OA Switchboard [129,130], the Open Journals System [131], Ubiquity Press [132], Scottish Universities Press [133] and Open Monograph Press. The Open Access Directory (OAD; see [134]) offers an information service compiling factual lists about open access and the SciFree Journal Search Tool allows researchers to search for journals offering open access publication.

Open research has also brought revolutions to journals, peer review and publishing models. Formed in 2016, Peer Community In (PCI) [135,136] is a non-for-profit, non-commercial platform that outsources and publishes the peer review of preprints and offers publication in their free open access journal. Another initiative named Peer Community In Registered Reports (PCI-RR; see [36,137–139]) was launched in 2021 and is dedicated to receiving, reviewing and recommending RRs via preprint servers. Funding is now available through Registered Reports Funding Partnerships [140,141] whereby funders and journals partner together in order to integrate their procedures for funding applications and RR submissions into one streamlined process; for example, Cancer Research UK require that if a funding application is successful, authors then submit their proposed research as a RR to one of 12 journals that are currently taking part in this pilot.

PeerRef also operates journal-independent peer-review through article preprints, aiming to make research assessment open, efficient and researcher-centric (see [135]). The journal F1000Research [142] started its journey in 2012, offering an open access platform that provides immediate publishing for articles with no editorial bias. Once a paper is deposited, expert reviewers are invited to perform transparent post-publication peer review, and their reports and names are published alongside the article together with the author’s responses and comments from registered users. Authors are then encouraged to publish revised versions of their article, with those that pass peer review indexed in external databases such as PubMed and Scopus. The Journal of Open Research Software (JORS) [143] and the Journal of Open Source Software (JOS) [144] publish software meta-papers and accompanying software packages, allowing recognition of the pivotal auxiliary outputs of research. Some journals, such as the Journal of Health Psychology, now have a mandatory data sharing policy (see [145]) which requires authors to make all raw data fully accessible to increase the transparency, openness and replicability of psychological research. The Peer Reviewer’s Openness (PRO) Initiative [146] is an initiative for peer reviewers themselves that declares a minimum requirement for publication of any scientific results must be the public submission of materials used in generating those results; signatories of this initiative will not offer comprehensive review for, nor recommend the publication of, any manuscript that does not meet these minimum requirements.

For open research to become normative, sustained and permanent, institutions and research organizations need to recognize, incentivize and reward it. Promisingly, initiatives are being implemented to achieve this. The San Francisco Declaration on Research Assessment (DORA) [147,148] is a worldwide initiative with the mission to advance practical and robust approaches to research assessment globally across all scholarly disciplines. In this light, DORA has worked globally with researchers, funders, institutions, learned societies and publishers to raise awareness of the need for research assessment reform, to discover and disseminate good practice, and to co-create new tools and processes that will enable real and positive change. To date, over 3000 organizations across 165 countries have signed up to this declaration [50], with positive changes in revised standards for hiring, promotion and progression highlighted by ReformScape [85,149], a searchable collection of criteria and standards for hiring, review, promotion and tenure from academic institutions. The Hong Kong Principles (HKPs) [150] help research institutions to minimize perverse incentives that can drive researchers to engage in QRPs by assessing responsible research practices, valuing complete reporting, rewarding open research practices, and acknowledging and recognizing research activities and tasks such as peer review and mentoring. Like DORA, the HKPs highlight issues with quantitative metrics such as publication impact factor or citation counts, while additionally outlining how such metrics are inappropriate for evaluating rigour and public involvement in research [151]. The Roundtable on Aligning Incentives for Open Science [152] convenes critical stakeholders to discuss the effectiveness of current incentives for adopting open research practices, current barriers and ways to move forward to optimally align reward structures and institutional values. The Contributor Roles Taxonomy (CRediT) [153] is a high-level taxonomy of roles that describe each contributor’s specific contribution to a scholarly output, shifting the traditional concept of authorship, and its associated rewards, to ensure that all those who make substantial contributions to a project are credited. Incentives to increase the adoption of open research practices have been developed, such as Open Science Badges [154,155] that acknowledge when preregistration, open materials and open data have been implemented. Open Research Awards (see [156]) are being led by many institutions and organizations to recognize researchers adopting open research practices or facilitating positive research culture reform.

### 3.4. Community initiatives

Community initiatives foster teamwork, collaboration and discussion within the scientific community to increase awareness or uptake of open research practices. Many of these initiatives are spearheaded by students and early career researchers (ECRs) aiming to aid awareness and uptake of open research through supporting, promoting and community building. A total of 87 community-based initiatives were identified.

**Figure 4.**
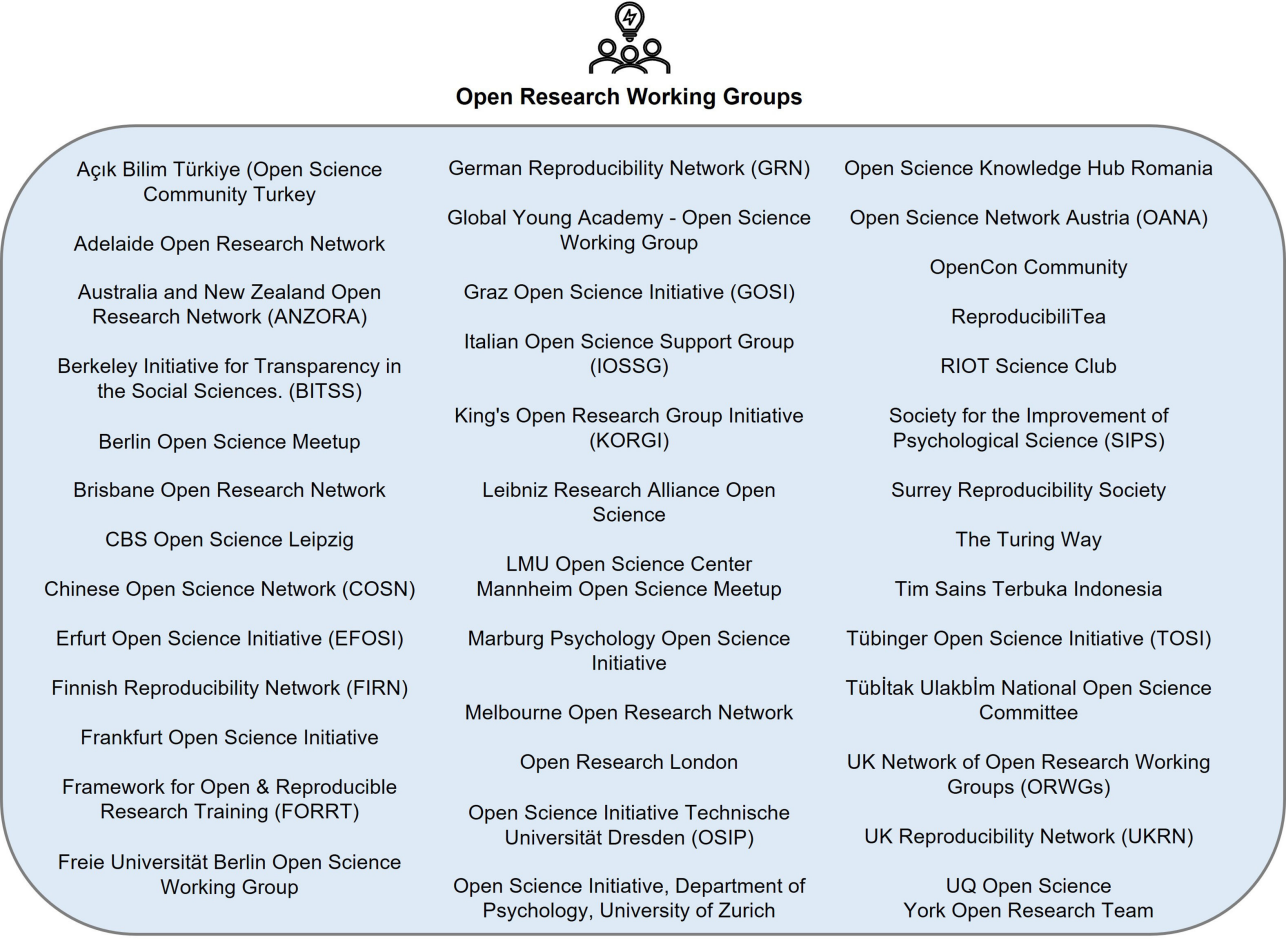
Overview of open research working groups, networks, societies, hubs and committees for researchers.

Most community initiatives were open research working groups, networks, societies, hubs and committees. These share common goals to discuss and advocate for awareness of open research, promote and organize training, and disseminate best practices. These communities are shown in figure 4 to aid brevity within the text. For example, the UK Reproducibility Network (UKRN) [59] is a national peer-led consortium that aims to promote and ensure rigorous research practices by establishing appropriate training activities, designing and evaluating research improvement efforts, disseminating best practice, and working with stakeholders to coordinate efforts across the sector. The volunteer community of UKRN have written several ‘primers’ on open research practices, such as preprints, preregistration and RRs. UKRN also coordinates the activities of numerous international reproducibility networks, with 19 countries currently affiliated, such as the German Reproducibility Network (GRN) [157] and the Finnish Reproducibility Network (FIRN) [158]. In addition, the UK Network of Open Research Working Groups (ORWGs) are action-oriented teams within higher education seeking to make the processes and products of research as transparent, accessible and reproducible as possible. They work together to develop policy initiatives, host events and conferences, produce educational materials and workshops, conduct collaborative research projects and assess community needs to bring more researchers towards open practices. The Berkeley Initiative for Transparency in the Social Sciences (BITSS) [159] aims to improve credibility of research through collaboration with researchers, faculty, students, publishers and funders to advance transparency, reproducibility, rigour and ethics in research. The Chinese Open Science Network (COSN) [160] raises awareness of open research through workshops, talks, journal clubs and resource translation. Some institutions also have open research teams, such as the York Open Research Team [161] who work with academic and research staff, postgraduate researchers and others to provide guidance and training in planning, publishing, preserving and sharing research. Similarly, the Tim Sains Terbuka (see [162]) aims to improve science and technology through open research in Indonesia.

There are also community initiatives that span multiple countries, institutions and organizations. Specifically, ReproducibiliTea (see [163,164]) is an initiative currently spanning across 113 institutions in 27 countries that helps researchers create local journal clubs at their universities to discuss papers and ideas centred around reproducibility, open science and scientific reform. Likewise, RIOT Science Club (see [163]) is a forum that aims to encourage ‘reproducible’, ‘interpretable’, ‘open’ and ‘transparent’ research allowing researchers to learn about, and keep up to date with new practices. The Turing Way [165] is a community-driven project that involves and supports a diverse community of contributors to make data science accessible, comprehensible and effective for everyone. They have guides for reproducible research, project design, communication, collaboration and ethical research. OpenDots is an initiative comprising international organizations, academic institutions, researchers and citizens, with the aim of creating a collaborative network that allows knowledge about open research to be concretized through information campaigns, workshops, webinars, open-source technology tools and platforms. The Society for the Improvement of Psychological Science (SIPS) [162] is an international organization that aims to improve the quality of methodology and practices in psychological research through training and community building, as well as contributing to the revision of institutional policies to incentivize better research practice.

For open research to be sustained, pedagogical reform is required through the integration of open and reproducible science into the taught curriculum. The Framework for Open and Reproducible Research Training (FORRT) [166] is a grassroots interdisciplinary and international community of over 1200 early career scholars dedicated to advancing open research through open education, pedagogical reform, social justice advocacy and meta-science. FORRT advocates for the integration of open research topics into higher education to advance research transparency, reproducibility, rigour and ethics, developing a wide range of OERs such as a glossary of open research terms, lesson plans and a series of community-built syllabi and teaching materials. By implementing initiatives designed to reduce barriers to participation in open research, FORRT actively works to democratize access to cutting-edge research practices and educational resources. These efforts ensure that underrepresented groups have the necessary tools, training and support to fully engage in transparent, rigorous and reproducible science, contributing to a more diverse and equitable global research community.

Other initiatives also share a common goal in highlighting the need for OERs for democratizing knowledge, reforming pedagogy and training students in open research practices. For example, The Research on Open Educational Resources for Development (ROER4D) [167] project investigates in what ways, and under what circumstances, the adoption of OERs can address the increasing demand for accessible, relevant, high-quality and affordable education in the Global South. The Collaborative Replications and Education Project (CREP) [168] provides training, support and professional growth opportunities for students and instructors completing replication projects. Promisingly, some community initiatives are driven by students themselves, such as the Student Initiative for Open Science (SIOS) [169] that focuses on educating social sciences undergraduates and graduates about responsible research practices with a particular emphasis on open research. The Carpentries (see [59]) builds global capacity in essential data and computational skills for conducting efficient, open and reproducible research by teaching foundational coding and data science skills to researchers worldwide. Sains Terbuka Airlangga is the first Indonesian initiative committed to promoting and educating students and young researchers to adopt open research practices. The Institute for Globally Distributed Open Research and Education (IGDORE) [134] is an independent research institute dedicated to improving science, education and quality of life for scientists, students and their families. The Higher Education Leadership Initiative for Open Scholarship (HELIOS) [170] is a cohort of colleges and universities committed to collective action to advance open scholarship within and across their campuses.

In addition to providing the necessary training for open research practices and providing a safe space for students, ECRs and researchers to discuss issues in the research ecosystem, there are initiatives whose goal is to specifically improve equality, representation, diversity and accessibility through wider research culture reform. Bullied Into Bad Science [134] aims to instigate institutions to take action to improve academic culture for ECRs and to create a fairer, more open and ethical research and publication environment. Free Our Knowledge [59] seeks a fairer and secure future in academia and a normalized open and reproducible research practice. FORRT also conducts targeted outreach to ECRs and scholars from low- and middle-income countries (LMICs) to foster a more equitable and diverse global research community.

Many community initiatives exist to foster citizen science, such as the Australian Citizen Science Association (ACSA) [171], the Citizen Science Association (CSA) [172], CoAct-Citizen Social Science [173], the European Citizen Science Association (ECSA) [174] and EU Citizen Science [175]. These communities seek to give citizen groups an equal ‘seat at the table’ through active participation in research and advance research progress through the sharing of knowledge, collaboration, capacity building and advocacy. The Code for Science and Society [176] is a not-for-profit organization that aims to improve the public’s ability to find, collect and share the open data they use to make more informed decisions in the benefit of public interest. The Open and Collaborative Science for Development Opportunities (OCSD) network [177] is a community of open research practitioners and leaders that learn together and contribute towards a pool of open knowledge on how collaboration could address local and global development challenges. Science communication podcasts that focus on open research have also been formed, such as the Everything Hertz podcast [178] that discusses methodology and scientific culture, and the ORION Open Science Podcast [179] that includes topics on data sharing, citizen science, peer review and professional development in open research.

Big team science has been advanced through the open science movement, which involves open, large-scale collaboration between researchers who work together to solve fundamental research questions and pool resources across different laboratories, institutions, disciplines, cultures and continents [180,181]. The Psychological Science Accelerator [182] is a globally distributed network of psychological science laboratories that coordinates data collection for democratically selected studies with the mission to accelerate the accumulation of reliable and generalizable research. The Many Labs initiative (e.g. [19,20]) accelerates big team science with a focus on replication studies, and has since joined hands with the initiative StudySwap [58], which is a platform for inter-laboratory replication, collaboration and research resource exchange. Supporting such initiatives, the repliCATS project [183] crowdsources predictions about the reliability and replicability of published research in social science fields. The Consortium for Reliability and Reproducibility (CoRR) [184] is yet another community-led initiative that has developed an open research resource for neuroimaging that facilitates the assessment of test–retest reliability and reproducibility of functional and structural connectomics studies through shared data. RedTeams (see [185]) work together to constructively criticize each other’s work or to find errors during the entire research process, with the overarching goal of maximizing research quality.

Several community-led repositories also aim to embed open research practices to improve research culture. These initiatives are categorized as community-based rather than structural because, although they provide new infrastructure for research, they are built and resourced by the scientific community *for* the scientific community. Some of these initiatives are associated with open access; for example, the Directory of Open Access Books (DOAB) [186] is a community-driven discovery service that indexes and provides access to scholarly, peer-reviewed open access books and helps users to find trusted open access book publishers, and PeerLibrary facilitates the global conversation on academic literature allowing users to share insights and exchange feedback to facilitate innovative research. Paperity is the first multidisciplinary aggregator of open access journals and papers, consolidating academia around open literature, and Unpaywall [187] is a free database of over 50 million open access scholarly articles. The Harvard Open Access Project [188] aims to facilitate the growth of open access through consultation, collaboration and community building and directs assistance to support research and policy analysis on open access. Some initiatives are dedicated to the ‘afterlife’ of published research articles, with a focus on rigorous and robust peer-review processes and research evaluation. Specifically, PREreview [134,135] is a Web platform for posting, reading and engaging with preprint reviews and The Unjournal [135] aims to build a better system for evaluating research through journal-independent feedback, ratings and evaluation of hosted papers. Furthermore, Qeios (see [135,189]) is a publishing platform that enables the open peer review of preprints, committed to fostering a research community that values open communication, rapid dissemination of knowledge and constructive feedback.

Other repositories have been built by the community to share datasets, software and research outputs. Zenodo [190] is a general-purpose open repository that allows researchers to deposit papers, datasets, software and digital artefacts, and rOpenSci [134] is a community initiative that aims to transform research through open data, software and reproducibility by developing R packages via community-driven learning, review and maintenance. Other initiatives promote knowledge dissemination more broadly. The Knowledge Futures Group [191] builds and supports products and protocols to make knowledge open and accessible to all; the Open Knowledge Foundation applies open knowledge to design infrastructures and organizations of the future; and the Open Scholar Community Interest Company develops ideas and tools that promote open and transparent research collaboration. Furthermore, LIBSENSE [192] is a programme aimed at building a community of practice and progressing adoption of open research services and infrastructures in Africa; FORCE11 [120] is a community of scholars, librarians, archivists, publishers and funders whose goal is to facilitate change through improved knowledge creation and sharing, the Goettingen Open Source and Science Initiative of Psychology (GOSSIP) [134] is a community committed to trustworthy and replicable results as well as the free availability of scientific results who hold regular information events and workshops on open science; and the Open Digital Health Initiative [193] is an organization that encourages health scientists, practitioners and technology developers to share evidence-based digital health tools.

Finally, some initiatives bring together communities of researchers dedicated to providing reliable open scholarly infrastructure through their joint efforts. Just One Giant Lab [194] provides a platform for open communities across the world to build impactful projects and offer special services for communities and organizations who require further guidance. The Joint Roadmap for Open Science Tools (JROST) [195] brings together key technology organizations and researchers who are actively involved in design and production of open scholarly infrastructure, offering workshops and other coordinated activities. Open Innovation in Science (OIS) [196] investigates and experiments with open and collaborative practices to generate new research questions and translating research into innovation.

## 4. Discussion

Open research reflects the idea that scientific knowledge of all kinds, where appropriate, should be accessible, transparent, rigorous, reproducible, replicable, accumulative and inclusive [29]. This systematic mapping review identified 187 international initiatives that aim to enhance awareness and uptake of open research practices in psychology, with each categorized into procedural (*n* = 30), structural (*n* = 70) and community-based change (*n* = 87). Although we focused on the discipline of psychology to guide this review, the initiatives identified are of relevance, and can improve research culture, across disciplines.

Procedural initiatives encompass behaviours and sets of commonly used practices in the research process and comprise toolkits, resources and guidelines for implementing open research, as well as the necessary infrastructure to support these. There are now numerous guides that teach students and researchers how to implement open research practices, such as preprints, study preregistration, RRs, open materials, code, software and data, helping to demystify these practices and mitigate perceived misnomers to their implementation. One helpful aspect of many of these toolkits and resources is that they offer different entry levels to ease into open research, or to overcome barriers: for example, researchers new to the practice of preregistration, or who are facing tight time constraints in their research, can use AsPredicted.org which asks researchers to answer nine simple questions about their research design and analyses; any researcher who has designed a study, or has acquired ethical approval, should know the answers to these questions making this a relatively simple and pain-free task. Once a researcher feels acquainted with this process, they can ‘level up’ to more extensive and detailed preregistration protocols, such as those offered on the Open Science Framework. Similarly, after trying their hand with preregistration, researchers can implement RRs within their research workflow: a publishing format that integrates study preregistration through a Stage 1 protocol and, upon receiving in principle acceptance, guarantees publication of the research so long as it meets the RR criteria. Some researchers argue that the uptake of open research has been slow. However, the scale of the initiatives identified within this review, which have been developed by researchers themselves and usually in a voluntary capacity, suggests there has been remarkable progress to integrate open research in psychology and beyond.

Traditionally, research practice has been governed by what are now understood to be problematic incentives arguably made normative by research institutions, organizations, publishers and funders. For example, many journals and research evaluation exercises have focused on the novelty of study findings and many funders focus on ‘blue sky’ or high-risk-high-reward ideas. Novel findings, of course, are important to accelerate scientific knowledge, but they need to be underpinned by rigorous, robust and transparent processes. Replications of research and reproducibility checks should also be recognized as equally, if not more, important. Researchers’ esteem has also routinely been recognized (i.e. hiring and promotion) through the quantity of their outputs or questionable metrics (e.g. citation h-index; journal impact factors) rather than research quality (e.g. robustness, rigour, transparency and inclusiveness). It is therefore promising to see vast changes in this sphere, too: our review identified 70 structural-based changes such as open research agendas, policies, frameworks and supporting infrastructure developed with the goal to make open research routine and normative. Notably, many government officials have recognized the requirement for open research to be at the core of the scientific enterprise, with the White House Office of Science and Technology Policy declaring 2023 the ‘Year of Open Science’, and the EUA developing an open science agenda. Indeed, strategies of behaviour change (see [51,52]) in this area propose that for open research to become sustained it needs to be made possible, easy, normalized, rewarded and required, with the latter influenced by such top-down structural initiatives. Promisingly, these initiatives have substantial backing from the research community: for example, over 7000 individuals and 1600 organizations have signed the Budapest Open Access Initiative declaration which aims to make research free and unrestricted in all academic fields internationally [197]; over 5000 journals and organizations have signed the TOP guidelines as a widely used tool for implementing open science practices (see https://osf.io/y2rr6) [116]; and over 25 000 individuals and organizations across 65 countries have signed DORA [147,148] to change the culture of research assessment.

Most open research initiatives, however, have been developed by bottom-up communities of students and ECRs passionate about changing the research landscape. This review identified 80 community-based initiatives, such as open research groups (e.g. UKRN [59]) and open scholarship communities that aim to embed the teaching of open research into the educational curriculum (e.g. FORRT [166]). Indeed, many of these initiatives, such as FORRT and ReproducibiliTEA, which rely on volunteer contributions and community-building, have demonstrated substantial impact in promoting open research education, meta-research, big team science and research integrity. The positive social element to these communities is invaluable in creating an open and non-judgemental space to discuss research culture, which reduces barriers to its implementation. Notably, the ReproducibiliTea journal club [164] helps researchers to create open research communities that discuss papers, ideas and issues relating to research. This entirely volunteer-based initiative is now implemented by researchers from over 113 institutions in 27 countries. Similarly, initiatives such as Bullied into Bad Science [134] and Free Our Knowledge [59] aim for a fairer, open and more ethical research landscape. Community initiatives therefore foster inclusion, teamwork and collaboration within the scientific community. To increase knowledge of open research practices more widely, there are also numerous podcasts such as Everything Hertz [178] and the ORION Open Science Podcast [179]. In order to increase collaboration, inclusiveness and representation in psychological research, the idea of big team science has also been advanced (see [180,181]), with community initiatives such as the Psychological Science Accelerator (PSA) [182] and StudySwap [58] providing better access to resources, allowing more diverse data to be collected and accelerating the accumulation of reliable and generalizable knowledge. Importantly, there has also been a drive to involve citizens directly in scientific research with initiatives such as the Citizen Science Association (CSA) [172] and European Citizen Science Association (ECSA) [174].

Together, then, numerous procedural, structural and community-based initiatives are enhancing both research practice and wider culture, contributing to the discipline of psychology becoming a trailblazer in open research.

### 4.1. Challenges and opportunities for sustaining open research

This systematic review identified 187 initiatives to enhance awareness and uptake of open research in psychology, highlighting extremely positive changes. To ensure that open research is sustained, however, several existing issues need to be addressed. First, it is essential that open research is normalized through coordinated and collaborative efforts between individuals, research groups, journals, funders, institutions and research organizations (see also [198]). If one element is addressed without the other (e.g. researchers focus on high-quality outputs (individual level) but are incentivized to focus on novelty (e.g. structural level)), then the problems we have seen historically will prevail and meaningful reform will fail [199]. Many community-led efforts are voluntary in nature and require support, recognition and funding. Indeed, there are encouraging developments in this sphere, such as new funding for meta-research and responsible research practice (e.g. UKRI, NWO, Research England, Einstein Foundation, SIPS) but many of these offer a limited amount of funding (compared with discipline-specific research), and this needs to be increased and sustained. Similarly, it is important that open research *initiatives* are developed and implemented in a collaborative and coordinated fashion to ensure that efforts are not duplicated and to avoid fragmentation. We identified many initiatives that appear to have common goals but exist separately within the research ecosystem: by joining these initiatives up and working together, their momentum and impact will likely be maximized. A promising example of such coordination comes from a partnership between FORRT and COS; FORRT’s curated resources database was increased by over 60% through integration with the COS’s Open Science Knowledge Base, and together these organizations are continually updating and validating a Replication Database.

Furthermore, this review highlights international initiatives demonstrating that a move to open research is widespread. However, there are geographical and regional gaps in open research and its associated initiatives, which reflects wider inequalities in support, funding and infrastructure for (open) research. For example, Li *et al*. [200] report that, in 2021, there were over 1000 open data repositories in the USA, 400 in Germany and 300 in the UK, yet only 48 in China. Furthermore, there are clear differences in the availability of resources by geographic region and between social groups, which present barriers to open research (see [44,45,52,201,202] for discussions). Indeed, some of the aforementioned initiatives aim to overcome such inequalities by sharing resources and funding (e.g. StudySwap, PSA) and facilitating research in—and with researchers from—underrepresented countries [47,48]. Such inequalities are imperative to discuss and mitigate in the context of open research, as they not only limit the global reach and impact of scientific advancements but also risk perpetuating systems where research from underrepresented regions and groups is marginalized [52]. Addressing these disparities through equitable access to resources, inclusive collaboration and targeted funding is required for creating a truly open, diverse and innovative research community that benefits all.

Furthermore, to sustain this database of initiatives itself, FORRT is currently developing an interactive, crowdsourced and living map of open research networks across disciplines to provide researchers with a detailed landscape of resources, opportunities, collaborations and initiatives across communities. This next initiative aims to reduce the aforementioned barriers by creating a centralized, openly accessible map that makes it easy for individuals and organizations to find and connect with relevant open research communities. FORRT is open to collaborations and people can submit their interest to participate.

## 5. Conclusion

The last decade has seen wide-scale behaviour change to encompass open research—a move to ensure knowledge is accessible, transparent, rigorous, reproducible, replicable, accumulative and inclusive. This review identified 187 procedural, structural and community-based initiatives that aim to enhance awareness and uptake of open research. The scale and momentum of these developments present an optimistic future for psychological science and beyond: through coordinated efforts between researchers, institutions, funders, journals, organizations and stakeholders, open research can lead to a more credible and useful research landscape, as well as a more inclusive, representative and diverse research culture. We hope that by compiling these many initiatives, this review promotes their further adoption and, through coordination, leads to complementary initiatives to sustain open research. We have made the resources underpinning this review publicly available to facilitate future evaluation of these initiatives’ effectiveness and impact.

## Data Availability

The PRISMA 2020 checklist, screening, article records and supporting materials are available on OSF [203]. Supplementary material is available online [204].

## Footnotes

^1^
The ‘replication crisis’ within psychology is also referred to as a ‘credibility revolution’ [2], renaissance [3] and opportunity/debate [4]. We use the term ‘crisis’ consistently through this article in line with Hussey [5] who suggests that crises are ‘a call to action [...] an urgency that motivates people to act’.
